# Effective Multifocus Image Fusion Based on HVS and BP Neural Network

**DOI:** 10.1155/2014/281073

**Published:** 2014-02-06

**Authors:** Yong Yang, Wenjuan Zheng, Shuying Huang

**Affiliations:** ^1^School of Information Technology, Jiangxi University of Finance and Economics, Nanchang 330013, China; ^2^School of Life Science and Technology, University of Electronic Science and Technology of China, Chengdu 610054, China; ^3^School of Software and Communication Engineering, Jiangxi University of Finance and Economics, Nanchang 330013, China

## Abstract

The aim of multifocus image fusion is to fuse the images taken from the same scene with different focuses to obtain a resultant image with all objects in focus. In this paper, a novel multifocus image fusion method based on human visual system (HVS) and back propagation (BP) neural network is presented. Three features which reflect the clarity of a pixel are firstly extracted and used to train a BP neural network to determine which pixel is clearer. The clearer pixels are then used to construct the initial fused image. Thirdly, the focused regions are detected by measuring the similarity between the source images and the initial fused image followed by morphological opening and closing operations. Finally, the final fused image is obtained by a fusion rule for those focused regions. Experimental results show that the proposed method can provide better performance and outperform several existing popular fusion methods in terms of both objective and subjective evaluations.

## 1. Introduction

Due to a finite depth of field of optical lenses, it is usually impossible to get an image in which all relevant objects are in focus; that is, only those objects within the depth of field of the camera will be in focus, while other objects will be out of focus [1]. Consequently, in order to obtain an image with every object in focus, images taken from the same scene focusing on different objects need to be fused, that is, multifocus image fusion [2]. Image fusion refers to an image preprocessing technique that combines two or more source images that have been registered into a single image according to some fusion rules. Its aim is to integrate complementary and redundant information of multiple images coming from the same scene to form a single image that contains more information of the scene than any of the individual source images [3]. Multifocus image fusion is an important branch of this field. The fused image obtained then turns out to be more suitable for human/machine perception, segmentation, feature extraction, detection, or target recognition tasks [4].

Image fusion is generally performed at different levels of information representation, namely, pixel level, feature level, and decision level [5]. Up to now, many multifocus image fusion techniques have been developed. Basically, the fusion technique can be categorized into spatial domain fusion and transform domain fusion [6]. The spatial domain-based methods directly select the clearer pixels or regions from source images in the spatial domain to construct the fused image [7, 8]. The basic idea of the transformed domain-based methods is to perform certain multiresolution decomposition on each source image, then integrate all these decompositions to obtain one combined representation according to some fusion rules, and finally reconstruct the fused image by performing the inverse transformation to the combined representation [9].

The simplest fusion method is to take the average of the source images pixel by pixel. The method is simple and suitable for real-time processing. However, it does not consider the correlation between the surrounding pixels and often leads to several undesired side effects such as reduced contrast [3]. In order to improve the quality of the fused image, the block-based multifocus image fusion methods have been proposed [7, 8]. These methods are shift-invariant, and all of the operations are performed in the spatial domain, so they have high computational efficiency. However, they are also faced with some problems. The first problem is how to determine the suitable size of the subblock. These methods usually suffer from block effects which severely reduce the quality of the fused image if the size of the subblock is selected unreasonably. Another problem is that which evaluation criteria would be more suitable to measure the clarity of the subblocks. In recent years, various approaches based on multiscale transforms have been proposed, including pyramid transform and wavelet transform, such as the Laplacian pyramid [10], gradient pyramid [11], the ratio of low pass pyramid [12], discrete wavelet transform (DWT) [13–15], shift-invariant discrete wavelet transform (SIDWT) [16], curvelet transform [17], contourlet transform [18], and nonsubsampled contourlet transform (NSCT) [19]. Pyramid decomposition-based image fusion can achieve a good effect. However, the pyramid decomposition of the image is redundant decomposition. The information of the different decomposition layers is correlative, which makes it easy to reduce the stability of the algorithm. Generally, DWT is superior to the previous pyramid-based methods because of providing directional information and without carrying redundant information across different resolutions. Moreover, DWT has good locality of time frequency. However, these methods based on multiscale transforms are shift-variant; namely, their performance will quickly deteriorate when there is a slight camera/object movement or there is misregistration of the source images [7, 20]. Although the SIDWT [16] and NSCT [19] algorithms both can overcome the shortcoming mentioned above, the implementation of the algorithm is more complicated and more time-consuming. Besides, some information of the source images may be lost during the inverse multiresolution transform implementation [21]. Recently, pulse coupled neural network (PCNN) has also been introduced to the multifocus image fusion, as seen in literature [22, 23]. However, the PCNN technique is very complex and has too many parameters. In addition, it is long and time-consuming.

In order to overcome the shortcoming of the methods mentioned above, in this paper, we propose a pixel level multifocus image fusion method based on HVS and BP neural network. Firstly, three features including texture feature, local visibility, and local visual feature contrast are extracted based on HVS and are used to train the BP neural network. Secondly, the initial fused image is acquired using BP neural network followed by a consistency verification process. Then, in order to avoid yielding any artificial or erroneous information that may be introduced during the process of preliminary fusion, the focused regions in each source image are determined by a hybrid procedure. Finally, the fused image is obtained based on the focused regions and initial fused image. The experiments show that the performance of the proposed method is superior to several existing fusion methods.

The rest of the paper is organized as follows. The related theory of the proposed method is described in Section 2. The fusion method that is based on HVS and BP neural network is introduced in Section 3. Experimental results and performance analysis are presented and discussed in Section 4, and the last section gives some concluding remarks.

## 2. Related Theoretical Knowledge

### 2.1. BP Neural Network

BP neural network is a multilayer feed-forward neural network, which is one of the most widely used neural networks. The problem of multifocus fusion based on BP neural work can be considered as a classification problem, focused or blurred.

**Figure 1:**
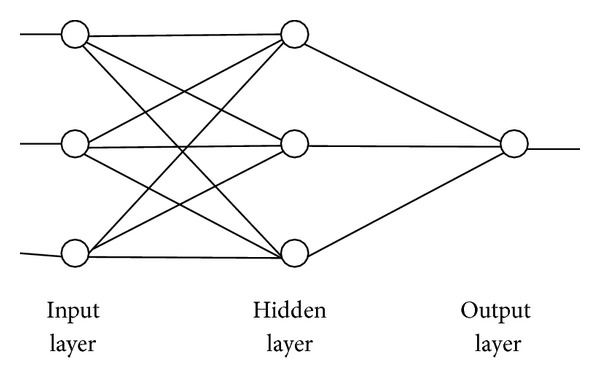
Architecture of BP neural network.

The basic BP neural network is a three-layer network, including input layer, hidden layer, and output layer. The architecture of BP neural network in the paper is shown in Figure 1. According to [24], we also adopt empirical formula to determine the number of nodes of the hidden layer, and the formula is defined as follows:

(1) $$\begin{array}{c} n_{h}=\text{sqrt}\left(n_{i}+n_{o}\right)+1, \end{array}$$

where *n*_*h*_, *n*_*i*_, and *n*_*o*_ are the number of nodes of the hidden layer, the number of nodes of the input layer, and the number of nodes of the output layer, respectively.

### 2.2. Features Extraction

In this paper, for each pixel, we extract three features based on the pixel centered of the 3 × 3 window to reflect its clarity. These are the texture features, local visibility, and local visual feature contrast.

#### 2.2.1. Texture Features

Log-Gabor filter was designed in the log coordinate system which is more conducive to the texture feature extraction [25]. The main advantage of the log-Gabor functions is that it can construct filters with arbitrary bandwidth under the condition of maintaining the DC component 0, which reduces filters redundancy. Furthermore, log-Gabor filters are more in line with the HVS. Texture features (TF) based on amplitude information reflect the high and low frequency energy distribution of the images. Therefore, taking the advantages of the log-Gabor filters into account, texture features of the multifocus image based on amplitude information will be extracted using log-Gabor filters. 2D Log-Gabor filter is defined in the frequency domain as follows [26]:

(2) $$\begin{array}{c} H\left(f,\theta \right)=H_{f}\times H_{\theta }, \end{array}$$

where *H*_*f*_ is radial component and *H*_*θ*_ is direction components. Specifically, the expressions are as follows:

(3) $$\begin{array}{c} H_{f}=\exp\left\{\frac{-{\left[\log(f/f_{0})\right]}^{2}}{2{\left[\log(\sigma_{f}/f_{0})\right]}^{2}}\right\},\\ H_{\theta }=\exp\left\{-\frac{{\left(\theta -\theta_{0}\right)}^{2}}{2\sigma_{\theta }^{2}}\right\}, \end{array}$$

in which *f*_0_ is the center frequency of filters, *θ*_0_ is the direction of filters, and *σ*_*f*_ is a constant that controls radial filters bandwidth *B*_*f*_. Consider

(4) $$\begin{array}{c} B_{f}=2\sqrt{\frac{2}{\log2}}\times \left|\log\left(\frac{\sigma_{f}}{f_{0}}\right)\right|. \end{array}$$

In order to obtain log-Gabor filters with the same bandwidth, *σ*_*f*_ must be changed along with *f*_0_ so that the value of *σ*_*f*_/*f*_0_ is constant. *σ*_*θ*_ determines direction bandwidth *B*_*θ*_. Consider

(5) $$\begin{array}{c} B_{\theta }=2\sigma_{\theta }\sqrt{2\log2}. \end{array}$$

#### 2.2.2. Local Visibility

In the paper, we introduce the concept of the image visibility (VI), which is inspired from the HVS and defined as follows [27]:

(6) $$\begin{array}{c} \mathrm{VI}=\frac{1}{M\times N}\overset{M}{\underset{i=1}{\sum }}\;\overset{N}{\underset{j=1}{\sum }}{\left(\frac{1}{m_{k}}\right)}^{\alpha }\times \frac{\left|I\left(i,j\right)-m_{k}\right|}{m_{k}}, \end{array}$$

where  *m*_*k*_ is the mean intensity value of the image, *α* is a visual constant ranging from 0.6 to 0.7, and *I*(*i*, *j*) denotes the gray value of pixel at position (*i*, *j*).

VI is more significant in multifocus image fusion than different sensor image fusion and the measurement has been successfully used in multifocus image fusion [27]. In the paper, in order to represent the clarity of a pixel, the local visibility (LVI) in spatial domain is proposed. The LVI is defined as

(7) $$\begin{array}{c} \text{LVI}\left(x,y\right)=\{\begin{array}{cc} \frac{1}{\left(2m+1\right)\times \left(2n+1\right)}\overset{m}{\underset{i=-m}{\sum }}\;\overset{n}{\underset{j=-n}{\sum }}{\left(\frac{1}{\bar{I}(x,y)}\right)}^{\alpha }\times \frac{\left|I\left(x+i,y+j\right)-\bar{I}\left(x,y\right)\right|}{\bar{I}\left(x,y\right)} & \text{if}\;\;\bar{I}\left(x,y\right)\neq 0\\ I\left(x,y\right) & \text{otherwise}, \end{array} \end{array}$$

where (2*m* + 1)×(2*n* + 1) is the size of neighborhood window and $\bar{I}(x,y)$ is the mean intensity value of the pixel (*x*, *y*) centered of the (2*m* + 1)×(2*n* + 1) window.

#### 2.2.3. Local Visual Feature Contrast

The findings of psychology and physiology have shown that HVS is highly sensitive to changes in the local contrast of the image, but insensitive to real luminance at each pixel [28]. The local luminance contrast formula is defined as follows:

(8) $$\begin{array}{c} C=\frac{L-L_{B}}{L_{B}}=\frac{\Delta L}{L_{B}}, \end{array}$$

where *L* is the local luminance and *L*_*B*_ is the local luminance of the background, namely, the low frequency component. Therefore, Δ*L* can be taken as the high frequency component. However, the value of single pixel is not enough to determine which pixel is focused without considering the correlation between the surrounding pixels. Therefore, to represent the salient features of the image more accurately the local visual feature (LVC) contrast in spatial domain is introduced, and is defined as

(9) $$\begin{array}{c} \text{LVC}\left(x,y\right)=\{\begin{array}{cc} {\left(\frac{1}{\bar{I}(x,y)}\right)}^{\alpha }\times \frac{\text{SML}\left(x,y\right)}{\bar{I}\left(x,y\right)} & \text{if}\;\;\bar{I}\left(x,y\right)\neq 0\\ \text{SML}\left(x,y\right) & \text{otherwise}, \end{array} \end{array}$$

where $\bar{I}(x,y)$ is the mean intensity value of the pixel (*x*, *y*) centered of the neighborhood window, *α* is a visual constant ranging from 0.6 to 0.7, and the SML(*x*, *y*) denotes the sum-modified-Laplacian (SML) located at (*x*, *y*), and more details about SML can be found in [7].

## 3. The Proposed Multifocus Image Fusion Method

### 3.1. Initial Fused Image Obtained by BP Neural Network

**Figure 2:**
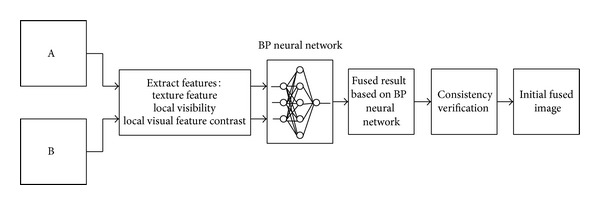
Schematic diagram of the BP neural network based fusion method.

Figure 2 shows the schematic diagram of the proposed method for obtaining the initial fused image based on BP neural network. Here, we only consider the case of two-source-image fusion, though the method can be extended straightforwardly to handle more than two, with the assumption that the source images have always been registered.

The algorithm first calculates salient features of each pixel form each source image by averaging over a small window. Assume that there are two pixels (one from each source image) and BP neural network is trained to determine which one is in focus. Then the initial fused image is constructed by selecting the clearer pixel followed by a consistency verification process. Specifically, the algorithm consists of the following steps.

**Step 1**

Assume that there are two source images *A* and *B*. Denote the *i*th pixel pair by *A*_*i*_ and *B*_*i*_, respectively.

**Step 2**

For each pixel, extract three features based on the pixel centered of the 3 × 3 window, which reflect its clarity (details in Section 2.2). Denote the feature vectors for *A*_*i*_ and *B*_*i*_ by (TF_*A*_*i*__, LVI_*A*_*i*__, LVC_*A*_*i*__) and (TF_*B*_*i*__, LVI_*B*_*i*__, LVC_*B*_*i*__), respectively.

**Step 3**

Train a BP neural network to determine which pixel is clearer. The difference vector (TF_*A*_*i*__ − TF_*B*_*i*__, LVI_*A*_*i*__ − LVI_*B*_*i*__, LVC_*A*_*i*__ − LVC_*B*_*i*__) is used as input, and the output is labeled according to

(10) $$\begin{array}{c} \text{targe}{\text{t}}_{i}=\{\begin{array}{cc} 1 & \text{if}\;\;A_{i}\;\;\text{is}\;\;\text{clearer}\;\;\text{than}\;\;B_{i},\\ 0 & \text{otherwise}. \end{array} \end{array}$$

**Step 4**

Perform simulation of the trained BP neural network on all pixel pairs. The *i*th pixel, *F*_*i*_, of the fused image is then constructed as

(11) $$\begin{array}{c} F_{i}=\{\begin{array}{cc} A_{i} & \text{if}\;\;\text{ou}{\text{t}}_{i}\geq 0.5\\ B_{i} & \text{otherwise}, \end{array} \end{array}$$

where out_*i*_ is the BP neural network output using the *i*th pixel pair as corresponding input.

**Step 5**

Verify consistency of the result of the fusion obtained in Step 4. Especially, when the BP neural network decides that a particular pixel is to come from *A* but with the majority of its surrounding pixel from *B*, this pixel will be changed to come from *B*.

### 3.2. The Method for Obtaining Final Fused Image

**Figure 3:**
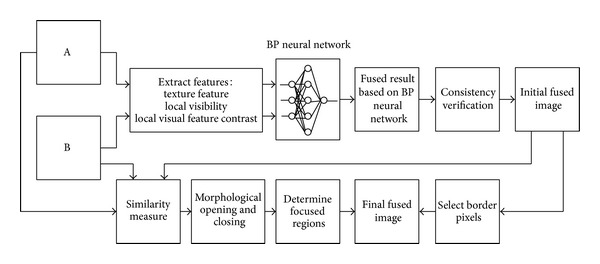
Schematic diagram of the proposed image fusion method.

In order to ensure that the pixels of the fused image come from the focused regions of each source image, we need to identify the focused regions in each source image firstly. Then the fused image can be constructed by simply selecting pixels in those regions. And as for the boundary of focused regions, the corresponding pixel of the initial fused image is selected as the pixel of the final fused image. Therefore, we proposed the following flow chart for obtaining the final fused image as illustrated in Figure 3.

#### 3.2.1. Detection of the Focused Regions

The pixels of the source images with higher similarity to the corresponding initial fused image pixels can be considered to be located in the focused regions. Thus, the focused regions in each source image can be determined by this method. In the paper, we adopt root mean square error (RMSE) [14] to measure the similarity between the source images and the initial fused image. Specifically, the algorithm of the detection of focused regions consists of the following steps.

**Step 1**

Calculate the RMSE of each pixel within (2*m* + 1)×(2*n* + 1) window between the source images and the initial fused image. Assume that *A* and *B* are two source images and *F* is the initial fused image. The formulas are defined as follows, respectively. In order to acquire the best fusion effect, we have tried different window sizes and found that the fusion effect is best when the size of the window is 5 × 5 or 7 × 7.

**Step 2**

Compare the values RMSE_*AF*_(*x*, *y*) and RMSE_*BF*_(*x*, *y*) to determine which pixel is in focus. The decision diagram, which is a binary image, will be constructed as follows:

(12) $$\begin{array}{c} Z\left(x,y\right)=\{\begin{array}{cc} 1 & \text{if}\;\;\text{RMS}{\text{E}}_{AF}\left(x,y\right)<{\text{RMSE}}_{BF}\left(x,y\right)\\ 0 & \text{otherwise}, \end{array} \end{array}$$

where “1” in *Z* indicates that the pixel at position (*x*, *y*) in source image *A* is in focus; conversely, the pixel in source image *B* is in focus, which indicates that the pixel with smaller RMSE(*x*, *y*) value is more possible in focus.

**Step 3**

In order to determine all the focused pixels and avoid the misjudgement of pixels, morphological opening and closing with small square structuring element and connected domain are employed. Opening, denoted as *Z*∘*b*, is that *Z* is eroded firstly by the structure element *b* followed by dilation of the result by *b*. It can smooth the contours of the object and remove narrow connections and small protrusions. Like the opening, closing can also smooth the contours of the object. However, the difference is that closing can join narrow gaps and fill the hole which is smaller than the structure element *b*. Closing is dilation by *b* followed by erosion by *b* and is denoted as *Z*•*b*. In fact, those small holes are usually generated by the misjudgement of pixels. What was worse, the holes larger than *b* are hard to remove simply using opening and closing operators. Therefore, a threshold TH should be set to remove the holes smaller than the threshold but larger than *b*. Then opening and closing are again used to smooth the contours of the object. Finally, the focused regions of each source image can be acquired, which can be more uniform and have well connected regions.

As for the structure element *b* and the TH, they can be determined according to the experimental results. In the paper, the structure element *b* is a 7 × 7 matrix with logical 1. In order to remove small and isolated areas which are misjudged, two different thresholds are set. The first threshold is set to be 20000 to remove areas which are focused in image *B* but misjudged as blurred. The second threshold is set to be 3000 to remove those areas which are focused in image *A* but misjudged as blurred.

#### 3.2.2. Fusion of the Focused Regions

The final fused image FF can be acquired according to the fusion rules that are as follows:

(13) $$\begin{array}{c} \text{FF}\left(x,y\right)=\{\begin{array}{cc} A\left(x,y\right) & \text{if}\;\;ZZ\left(x,y\right)==1,\;\;\text{count}\left(x,y\right)\\ & =\left(2m+1\right)\times \left(2n+1\right)\\ B\left(x,y\right) & \text{if}\;\;ZZ\left(x,y\right)==0,\;\;\text{count}\left(x,y\right)=0\\ F\left(x,y\right) & \text{otherwise}, \end{array} \end{array}$$

where

(14) $$\begin{array}{c} \text{count}\left(x,y\right)=\overset{i=m}{\underset{i=-m}{\sum }}\overset{j=n}{\underset{j=-n}{\sum }}ZZ\left(x+i,y+j\right), \end{array}$$

*ZZ* is the modified *Z* matrix of Step 3 in Section 3.2.1, *A*(*x*, *y*), *B*(*x*, *y*), *F*(*x*, *y*), and FF(*x*, *y*) denote the gray value of pixel at position (*x*, *y*) of the source images (*A* and *B*), the initial fused image *F*, and the final fused image FF, respectively, and (2*m* + 1)×(2*n* + 1) is the size of slipping window; count(*x*, *y*) = (2*m* + 1)×(2*n* + 1) suggests that the pixel at position (*x*, *y*) in image *A* is in focus and will be selected as the pixel of the final fused image FF directly. On the contrary, count(*x*, *y*) = 0 indicates that the pixel at the position coming from image *B* is focused and can be chosen as the pixel of the final fused image FF. Other cases, namely, 0 < count(*x*, *y*)<(2*m* + 1)×(2*n* + 1), imply that the pixel at position (*x*, *y*) is located in the boundary of focused regions, and the corresponding pixel of the initial fused image *F* is selected as the pixel of the final fused image FF.

## 4. Experimental Results and Performance Analysis

### 4.1. Experimental Setup

**Figure 4:**
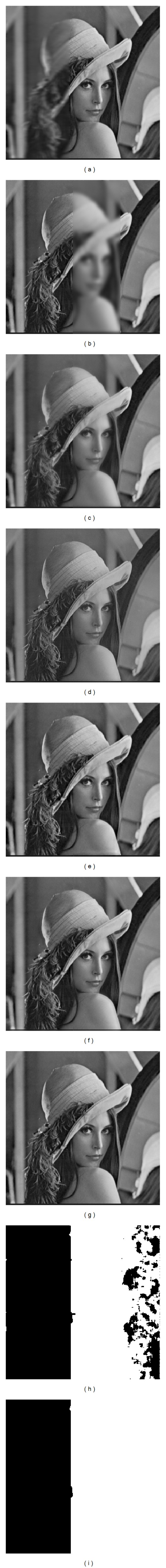
Original images and fused images of “lena”: (a) focus on the right; (b) focus on left and right sides; (c) fused image using average; (d) fused image using gradient pyramid; (e) fused image using DWT; (f) fused image using SIDWT; (g) fused image using the proposed method; (h) the initial focused region; (i) the modified focused region.

**Figure 5:**
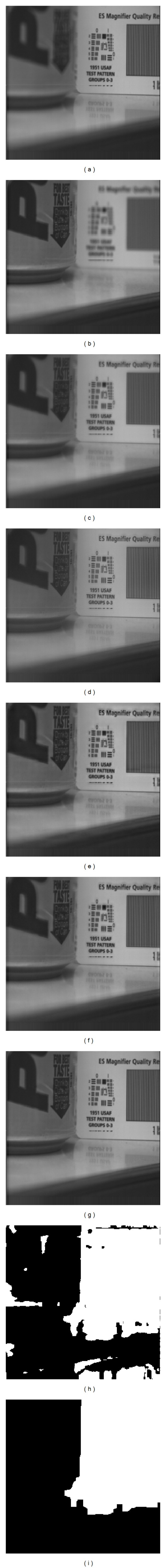
Original and fused image of “pepsi”: (a) focus on the right; (b) focus on the left; (c) fused image using average; (d) fused image using gradient pyramid; (e) fused image using DWT; (f) fused image using SIDWT; (g) fused image using the proposed method; (h) the initial focused region; (i) the modified focused region.

**Figure 6:**
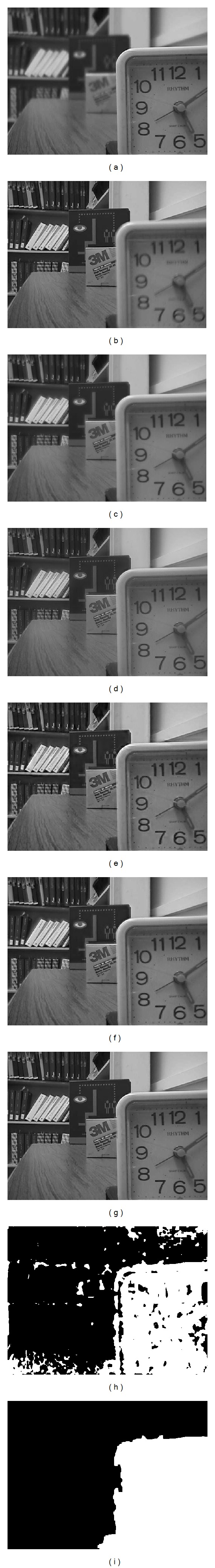
Original and fused image of “disk”: (a) focus on the right; (b) focus on the left; (c) fused image using average; (d) fused image using gradient pyramid; (e) fused image using DWT; (f) fused image using SIDWT; (g) fused image using the proposed method; (h) the initial focused region; (i) the modified focused region.

In this section, the first step we should do is to train the BP neural network. The training experiment is performed on the standard popular widely used “lena” image, which is a 256-level image with all in focus. We then artificially produce three out-of-focus images blurred with Gaussian radius of 0.5, 1.0, and 1.5, respectively. A training set with a total of 4 × 256 × 256 pixel pairs is formed. The three features of each pixel, TF, LVI, and LVC, are extracted with *α* = 0.65. In addition, we artificially produce a pair of out-of-focus images shown in Figures 4(a) and 4(b), which are acquired by blurring the left part and the middle part of the original image using the Gaussian function, respectively. To evaluate the advantage of the proposed fusion method, experiments are performed on three sets of source images as shown in Figures 4, 5, and 6, respectively, including one set of source images produced artificially and two sets of source images acquired naturally. Their sizes are 256 × 256, 256 × 256, and 640 × 480, respectively. These images all contain multiple objects at different distances from the camera and only those objects within the depth of field of the camera will be focused, while other objects naturally will be out of focus when taken. For example, Figure 5(a) is focused on testing card, while Figure 5(b) is focused on the pepsi can.

In order to compare the performance of the proposed fusion method, these multifocus images are also performed using the conventional and classical methods, such as taking the average of the source images pixel by pixel, the gradient pyramid method [11], the DWT-based method, and the SIDWT-based method [16]. The decomposition level of the multiscale transform is 4 layers. The wavelet basis of the DWT and SIDWT is DBSS (2,2) and Haar, respectively. The fusion rules of lowpass subband coefficients and the highpass subband coefficients are the “averaging” scheme and the “absolute maximum choosing” scheme, respectively.

### 4.2. Evaluation Criteria

In general, the evaluation methods of image fusion can be categorized into subjective methods and objective methods. However, observer personal visual differences and psychological factors will affect the results of image evaluation. Furthermore, in most cases, it is difficult for us to perceive the difference among fusion results. Therefore, the subjective evaluation of the fused results is always incomprehensive. Hence, in addition to the subjective evaluation, we also adopt several metrics to objectively evaluate the image fusion results and quantitatively compare the different fusion methods in the paper.

#### 4.2.1. Mutual Information (MI)

The mutual information MI_*AF*_ between the source image *A* and the fused image *F* is defined as follows:

(15) $$\begin{array}{c} \text{M}{\text{I}}_{AF}=\overset{L-1}{\underset{k=0}{\sum }}\;\overset{L-1}{\underset{i=0}{\sum }}p_{AF}\left(k,i\right)\text{lo}{\text{g}}_{2}\frac{p_{AF}\left(k,i\right)}{p_{A}\left(k\right)\times p_{F}\left(i\right)}, \end{array}$$

where *p*_*AF*_ is the jointly normalized histogram of *A* and *F*, *p*_*A*_ and *p*_*F*_ are the normalized histograms of *A* and *F*, *L* is the gray level of the image, and *k* and *i* represent the pixel value of the images *A* and *F*, respectively. The mutual information MI_*BF*_ between the source image *B* and the fused image *F* is similar to MI_*AF*_. The mutual information between the source images *A*, *B* and the fused image *F* is defined as follows:

(16) $$\begin{array}{c} \text{M}{\text{I}}_{F}^{AB}=\text{M}{\text{I}}_{AF}+\text{M}{\text{I}}_{BF}. \end{array}$$

The metric reflects the total amount of information that the fused image *F* contains about source images *A* and *B*. The larger the value is, the more the information is obtained from the original image and the better the fusion effect is.

#### 4.2.2. *Q* ^*AB*/*F*^

The metric *Q* ^*AB*/*F*^ evaluates the sum of edge information preservation values and is defined as follows:

(17) QAB/F=(∑m=1M ∑n=1N(QAF(m,n)×ωA(m,n)+QBF(m,n)×ωB(m,n)))×(∑m=1M ∑n=1N(ωA(m,n)+ωB(m,n)))−1,

where *Q* ^*AF*^(*m*, *n*) = *Q*_*g*_^*AF*^(*m*, *n*)*Q*_*α*_^*AF*^(*m*, *n*), *Q*_*g*_^*AF*^(*m*, *n*) and *Q*_*α*_^*AF*^(*m*, *n*) are the edge strength and orientation preservation values, respectively, *Q* ^*BF*^(*m*, *n*) is similar to *Q* ^*AF*^(*m*, *n*), and *ω*_*A*_(*m*, *n*) and *ω*_*B*_(*m*, *n*) are weights to measure the importance of *Q* ^*AF*^(*m*, *n*) and *Q* ^*BF*^(*m*, *n*), respectively, The dynamic range of *Q* ^*AB*/*F*^ is [0,1] and it should be as close to 1 as possible, and for the “ideal fusion”, *Q* ^*AB*/*F*^ = 1. In addition, (*m*, *n*) represents the pixel location, and *M* and *N* are the size of images, respectively.

The *Q* ^*AB*/*F*^ metric reflects the quality of visual information obtained from the fusion of input images. Therefore, the larger the value, the better the performance.

#### 4.2.3. Correlation Coefficient (CORR)

Correlation coefficient between the fused image *F* and the standard reference image *R* is defined as follows:

(18) CORR=(∑m=1M∑n=1N(R(m,n)−R¯(m,n))(F(m,n)−F¯(m,n))) ×(∑m=1M∑n=1N(R(m,n)−R¯(m,n))2×∑m=1M∑n=1N(F(m,n)−F¯(m,n))2)−1/2,

where $\bar{R}(m,n)$ and $\bar{F}(m,n)$ represent the pixel gray average value of the standard reference image *R* and fused image *F*, respectively.

The metric reflects the degree of correlation between the fused image and the standard reference image. The larger the value is, the better the fusion effect is.

#### 4.2.4. Root Mean Squared Error (RMSE)

Root mean square error (RMSE) between the fusion image *F* and the standard reference image *R* is defined as follows:

(19) $$\begin{array}{c} \text{RMSE}=\sqrt{\frac{\sum_{m=1}^{M}\sum_{n=1}^{N}{\left(R\left(m,n\right)-F\left(m,n\right)\right)}^{2}}{M\times N}}. \end{array}$$

The metric is used to measure the difference between the fused image and the standard reference image. The smaller the value is, the better the fusion effect is.

### 4.3. Fusion of Artificial Test Images

The experiment is performed on a pair of “lena” multifocus images as shown in Figures 4(a) and 4(b). The initial and modified detected focused regions are shown in Figures 4(h) and 4(i), respectively. The white pixels in Figure 4(i) indicate that corresponding pixels from Figure 4(a) are in focused regions, while the black pixels suggest that corresponding pixels from Figure 4(b) are in focused regions. By comparison, we can observe that the detected focused regions of Figure 4(i) are better than those of Figure 4(h); for example, there are some misdetected focused regions in the right side of Figure 4(h), whereas they are correctly detected in Figure 4(i) because the right side of it is almost totally white. The fusion results obtained by the previous five different methods are shown in Figures 4(c)–4(g), respectively. It can be found that the results of the pixel averaging and gradient pyramid method have a poor contrast compared to those of the DWT-based method, the SIDWT-based method, and the proposed method. However, it is difficult for us to perceive the difference among the results of the DWT-based method, the SIDWT-based method, and the proposed method according to the subjective evaluation. Therefore, to objectively evaluate these five fusion methods, quantitative assessments of the five fusion results are needed. The results of the quantitative assessments are shown in Table 1. As can be seen from Table 1, MI, *Q* ^*AB*/*F*^, and CORR values of the proposed method are higher and RMSE value is less than those of the other methods, which means that by using our proposed method, the best quantitative evaluation results have been achieved.

### 4.4. Fusion of Real Digital Camera Images

The experiments carried out in this section are performed on two sets of source images acquired naturally as shown in Figures 5(a)-5(b) and Figures 6(a)-6(b), respectively. The initial and modified detected focused regions of those two sets of source images are shown in Figures 5(h)-5(i) and Figures 6(h)-6(i), respectively. The fused images obtained by using pixel averaging method, gradient pyramid method, DWT-based method, the SIDWT-based method, and the proposed method on these two sets of source images are shown in Figures 5(c)–5(g) and Figures 6(c)–6(g), respectively. From the fusion results, we can easily observe that fusion effects acquired based on the pixel averaging and gradient pyramid are not satisfactory and with poor contrast. For example, the regions of the testing card in Figures 5(c)-5(d) are not clear, but they are clear in Figures 5(e)–5(g). But it is difficult to discriminate the difference among the results of the DWT-based method, the SIDWT-based method, and the proposed method by subjective evaluation, so we need to do objective evaluation. However, it should be noted that the reference image is usually not available for real multifocus images, so only the two evaluation criteria including the MI and *Q* ^*AB*/*F*^ are used to objectively compare the fusion results. The quantitative comparison of the five methods for fusion of these two sets of source images is shown in Tables 2 and 3, respectively. As can be seen from the two tables, we can find that the MI and *Q* ^*AB*/*F*^ values of the proposed method are significantly higher than those of the other methods. It should be noted that we have carried out experiments on other multifocus images, and their results are identical to these two examples, so we did not mention all of them here. Therefore, the results of subjective and objective evaluation presented here can verify that the performance of the proposed method is superior to those of the other methods.

## 5. Conclusions

By combining the idea of the correlation between the neighboring pixels and BP neural networks, a novel multifocus image fusion method based on HVS and BP neural network is proposed in the paper. Three features which are based on HVS and can reflect the clarity of a pixel are extracted and used to train a BP neural network to determine which pixel is clearer. The clearer pixels are combined to form the initial fused image. Then the focused regions are detected by judging whether pixels from the initial fused image are in the focused regions or not. Finally the final fused image is obtained with the help of the technique of focused region detection by a certain fusion rule. The results of subjective and objective evaluation of several experiments show that the proposed method outperforms several popular widely used fusion methods. In the future, we will focus on improving the robustness of the method for noise.

## Figures and Tables

**Table 1:** Performance comparison of different fusion algorithms in Figure 4.

Fusion algorithms	MI	*Q* ^*AB*/*F*^	CORR	RMSE
Average method	7.4946	0.72072	0.98987	8.8023
Gradient pyramid	5.102	0.72693	0.98876	13.791
DWT	7.1207	0.76948	0.99784	4.0532
SIDWT	7.3712	0.77171	0.99553	5.7595
Proposed method	9.8067	0.81128	0.99962	2.8699

**Table 2:** Performance comparison of different fusion algorithms in Figure 5.

Fusion algorithms	MI	*Q* ^*AB*/*F*^
Average method	7.2941	0.64922
Gradient pyramid	5.9768	0.67975
DWT	6.4442	0.68264
SIDWT	6.82160	0.70890
Proposed method	9.1957	0.75904

**Table 3:** Performance comparison of different fusion algorithms in Figure 6.

Fusion algorithms	MI	*Q* ^*AB*/*F*^
Average method	5.9845	0.52143
Gradient pyramid	5.3657	0.63792
DWT	5.3949	0.64323
SIDWT	5.8380	0.67620
Proposed method	8.3105	0.73806
